# Several factors that predict the outcome of large B‐cell lymphoma patients who relapse/progress after chimeric antigen receptor (CAR) T‐cell therapy can be identified before cell administration

**DOI:** 10.1002/cam4.70138

**Published:** 2024-09-09

**Authors:** Alice Sýkorová, František Folber, Kamila Polgárová, Heidi Móciková, Juraj Ďuraš, Kateřina Steinerová, Aleš Obr, Adriana Heindorfer, Miriam Ladická, Ľubica Lukáčová, Erika Čellárová, Ivana Plameňová, David Belada, Andrea Janíková, Marek Trněný, Tereza Jančárková, Vít Procházka, Andrej Vranovský, Margaréta Králiková, Jan Vydra, Lukáš Smolej, Ľuboš Drgoňa, Martin Sedmina, Eva Čermáková, Robert Pytlík

**Affiliations:** ^1^ 4th Department of Internal Medicine – Haematology University Hospital and Faculty of Medicine Hradec Králové Czech Republic; ^2^ Department of Internal Medicine, Haematology and Oncology Masaryk University Hospital Brno Czech Republic; ^3^ 1st Department of Medicine‐Department of Haematology Charles University, General University Hospital Prague Czech Republic; ^4^ Department of Haematology University Hospital Královské Vinohrady and Third Faculty of Medicine, Charles University Prague Czech Republic; ^5^ Department of Haemato‐oncology University Hospital Ostrava and Faculty of Medicine, University of Ostrava Ostrava Czech Republic; ^6^ Department of Haematology and Oncology University Hospital Pilsen Czech Republic; ^7^ Department of Haemato‐Oncology, Faculty of Medicine and Dentistry Palacky University Olomouc Czech Republic; ^8^ Department of Haematology Hospital Liberec Liberec Czech Republic; ^9^ Clinic of Oncohaematology Medical Faculty of Comenius University and National Cancer Institute Bratislava Slovakia; ^10^ Oncology Clinic J.A. Reiman Faculty Hospital Prešov Slovakia; ^11^ Department of Haematology F.D. Roosevelt University Hospital Banská Bystrica Slovakia; ^12^ Clinic of Haematology and Transfusion Medicine Jessenius Faculty of Medicine in Martin, Comenius University in Bratislava Martin Slovakia; ^13^ Institute of Haematology and Blood Transfusion Prague Czech Republic; ^14^ Department of Medical Biophysics, Faculty of Medicine in Hradec Kralove Charles University Hradec Kralove Czech Republic

**Keywords:** CAR T‐cell failure, outcomes of patients after CAR T‐cell therapy failure, relapsed/refractory large B‐cell lymphoma, risk factors for CAR T‐cell therapy failure

## Abstract

**Aim:**

The aim of this study was to analyse the outcomes of patients with large B‐cell lymphoma (LBCL) treated with chimeric antigen receptor T‐cell therapy (CAR‐Tx), with a focus on outcomes after CAR T‐cell failure, and to define the risk factors for rapid progression and further treatment.

**Methods:**

We analysed 107 patients with LBCL from the Czech Republic and Slovakia who were treated in ≥3rd‐line with tisagenlecleucel or axicabtagene ciloleucel between 2019 and 2022.

**Results:**

The overall response rate (ORR) was 60%, with a 50% complete response (CR) rate. The median progression‐free survival (PFS) and overall survival (OS) were 4.3 and 26.4 months, respectively. Sixty‐three patients (59%) were refractory or relapsed after CAR‐Tx. Of these patients, 39 received radiotherapy or systemic therapy, with an ORR of 22% (CR 8%). The median follow‐up of surviving patients in whom treatment failed was 10.6 months. Several factors predicting further treatment administration and outcomes were present even before CAR‐Tx. Risk factors for not receiving further therapy after CAR‐Tx failure were high lactate dehydrogenase (LDH) levels before apheresis, extranodal involvement (EN), high ferritin levels before lymphodepletion (LD) and ECOG PS >1 at R/P. The median OS‐2 (from R/P after CAR‐Tx) was 6.7 months (6‐month 57.9%) for treated patients and 0.4 months (6‐month 4.2%) for untreated patients (*p* < 0.001). The median PFS‐2 (from R/P after CAR‐Tx) was 3.2 months (6‐month 28.5%) for treated patients. The risk factors for a shorter PFS‐2 (*n* = 39) included: CRP > limit of the normal range (LNR) before LD, albumin < LNR and ECOG PS > 1 at R/P. All these factors, together with LDH > LNR before LD and EN involvement at R/P, predicted OS‐2 for treated patients.

**Conclusion:**

Our findings allow better stratification of CAR‐Tx candidates and stress the need for a proactive approach (earlier restaging, intervention after partial remission achievement).

## 1 INTRODUCTION

Patients with relapsed and refractory (R/R) diffuse large B‐cell lymphoma (DLBCL) have a poor prognosis.^1^, ^2^, ^3^ Chimeric antigen receptor T‐cell therapy (CAR‐Tx) represents a new, revolutionary treatment for R/R DLBCL patients.^4^, ^5^, ^6^, ^7^, ^8^, ^9^, ^10^ CAR T‐cells expressing an anti‐CD19 CAR have been tested in heavily (≥2 lines of treatment) pretreated DLBCL patients, with encouraging results. The overall response rates (ORRs) were 52%–83%, and the complete response (CR) rates were 40%–58%, with durable CR in 30%–40% of patients.^11^, ^12^, ^13^ Based on these studies, the commercial products tisagenlecleucel (tisa‐cel, Kymriah), axicabtagene ciloleucel (axi‐cel, Yescarta) and lisocabtagene maraleucel (liso‐cel, Breyanzi) were approved for the treatment of R/R DLBCL patients after failure of ≥2 lines of therapy.^11^, ^12^, ^13^, ^14^, ^15^, ^16^ With these successes in mind, we still need to acknowledge that the majority of patients treated with CAR‐Tx are not cured, and the prognosis of patients who progress after this therapy is very poor (median overall survival [OS] 5–6 months).^17^ Therefore, we need to enhance the efficacy of CAR‐Tx, develop tools to predict treatment failure and identify effective treatments for progressing patients. The causes of CAR‐Tx failure can be divided into patient‐related, tumour‐related and product‐related factors.^18^, ^19^, ^20^ Patient‐related factors include Eastern Cooperative Oncology Group Performance Status (ECOG PS), comorbidities, inflammatory status (C‐reactive protein [CRP], ferritin, interleukin‐6) and nutritional markers (albumin). Tumour‐related factors are divided into factors associated with tumour burden and intrinsic tumour factors.^21^, ^22^, ^23^, ^24^, ^25^, ^26^, ^27^, ^28^, ^29^, ^30^, ^31^ Product‐related factors are divided into intrinsic factors in the input material^32^ and factors in the final cellular product.^33^, ^34^, ^35^, ^36^, ^37^, ^38^ Previous treatment, especially treatment with bendamustine, can negatively affect the input material and final product quality.^39^ Some studies have confirmed the impact of bridging therapy administration, the type of bridging/lymphodepletion regimen before CAR‐Tx, the type of CAR T‐cell product and the impact of CAR T‐cell expansion on survival.^11^, ^40^, ^41^, ^42^ We need to expand upon the limited data on the predictive value of various risk factors for CAR T‐cell failure, and we need to better define the optimal management of progression. Our goal was to detect the failure of CAR‐Tx and deliver the appropriate treatment as soon as possible. Therefore, the aims of our study were as follows:

1. To analyse the outcomes of LBCL patients after CAR‐Tx with a focus on CAR T‐cell failure patients.
2. To define the risk factors for rapid progressive disease after CAR‐Tx that will limit one's ability to receive further active treatment.

### 1.1 PATIENTS

Since 2019, anti‐CD19 CAR‐Tx has been used as a treatment modality for R/R LBCL patients after ≥2 lines of treatment in the Czech Republic. All patients ≥18 years with R/R aggressive LBCL after ≥2 lines of treatment who were treated with anti‐CD19 commercial products (tisagenlecleucel [tisacel], axicabtagene ciloleucel [axicel]), and those who failed on this therapy from December 2019 to December 2022 were included in this analysis. Patients from Slovakia who were treated in the Czech Republic before CAR‐Tx was available in their country were also included, resulting in a total of 107 patients. Thirty one (29%) patients were heavily pretreated (≥4 lines of therapy for LBCL before apheresis for CAR‐Tx).

The data were retrospectively collected from the electronic medical records of each institution through chart review by the individual investigators.

## 2 METHODS

The histological diagnosis of aggressive LBCL was established according to the World Health Organisation Classification criteria of 2008.^43^ Chimeric antigen receptor T‐cell failure was defined as the best response of either stable disease or progression, relapse from complete remission, or progression after a best response of partial remission. Data on parameters at the time of apheresis (APH), before lymphodepletion (LD) and at the time of relapse/progression (R/P) were collected:

### 2.1 At the time of apheresis (APH)

Histological subtype of LBCL, age at APH, presence of the ‘bulky disease’ (≥5 cm) at APH, ECOG PS at APH, level of LDH at APH, presence of extranodal (EN)/central nervous system (CNS) involvement at APH; number of treatment lines before APH and bendamustine pretreatment.

### 2.2 Before lymphodepletion (LD)

LDH, ECOG PS, ‘bulky disease’ (≥5 cm), EN/CNS involvement, CRP, albumin and full blood count parameters, including lymphocyte count, the value of ferritin just before LD and on the day of CAR T‐cell administration (D0), the International Prognostic Index (IPI) + age‐adjusted (AA) IPI and the type of bridging therapy. We analysed the type of LD agent and available CAR T‐cell peak expansion data after CAR‐Tx.

### 2.3 At the time of R/P after CAR T‐cell failure

Whether a new biopsy of the tumour was taken, whether CD19 antigen was found in the biopsy, persistence of CAR T‐cells, ‘bulky disease’ (≥5 cm), ECOG PS, assessment of LDH and albumin values, EN/CNS involvement, clinical stage III–IV and IPI/AA IPI.

Lymphodepleting regimens were used as described in original publications.^11^, ^12^ The response was evaluated according to the Lugano classification.^44^, ^45^, ^46^ Treatment response was evaluated according to institutional standards (PET/CT or CT examination). The best treatment response after CAR‐Tx was documented. The median follow‐up was evaluated among surviving (the whole group and the subgroup with R/P after CAR‐Tx).

For the detection of CAR T‐cells in peripheral blood, a multicolour flow cytometry panel was used. For surface staining of T cells and their subpopulations, the following monoclonal antibodies were used: anti‐CD4 peridinin‐chlorophyll‐protein complex‐cyanine (5.5), anti‐CD19 R‐phycoerythrin‐cyanine7, anti‐CD3 allophycocyanin, anti‐CD8 allophycocyanin‐cyanine7, anti‐CD20 pacific blue and anti‐CD45 pacific orange. Then, an anti‐FMC63 scFv antibody (ACROBiosystems, Newark, DE, USA) conjugated with R‐phycoerythrin was used to detect of CAR T‐cells.

To calculate the survival of R/P patients after CAR‐Tx, we evaluated overall survival‐2 (OS‐2) (defined below) in the whole group (*n* = 63), in treated patients (*n* = 39, OS treated‐2) and in untreated patients (*n* = 24, OSuntreated‐2). Progression‐free survival‐2 (PFS‐2) (defined below) was evaluated only in treated patients (PFS treated‐2) because in untreated subjects, the date of the PFS‐2 event was the same as the date of progression after CAR‐Tx. The database was locked for the last follow‐up in August 2023. The study adhered to the Declaration of Helsinki.

### 2.4 STATISTICS

The data were analysed using the statistical software NCSS 2021 (NCSS, LLC, Kaysville, Utah, USA, ncss.com/software/ncs) and MedCalc software (Mariakerke, Belgium). Overall survival (OS) and progression‐free survival (PFS) curves were estimated with the Kaplan–Meier method, and differences were compared by the stratified log‐rank test.

Progression‐free survival was defined as the time from CAR T‐cell infusion (day 0) until the first R/P disease or death from any cause. OS was defined as the time from CAR T‐cell infusion (day 0) to documented death from any cause. OS‐2 was defined as the time from the diagnosis of R/P after CAR‐Tx to documented death from any cause. OS treated‐2 was defined as the time from diagnosis of R/P after CAR T‐cell infusion to documented death from any cause in patients who received active treatment for R/P after CAR‐Tx. PFS treated‐2 was defined as the time from R/P after CAR‐Tx to the second R/P after CAR‐Tx or death from any cause in patients who were treated for R/P after CAR‐Tx. Fisher's exact test was used to analyse differences between groups for categorical variables. The Mann–Whitney test was used for quantitative variables. Multivariate analysis was performed by Cox regression analysis using the hierarchic forward stepwise selection process. Multivariate logistic regression was used to identify risk factors for failure to undergo treatment at R/P after CAR T‐cell therapy. All point estimates are presented with an appropriate 95% confidence interval. The level of significance was *α* = 0.05. All *p* values were two‐sided.

## 3 RESULTS

### 3.1 Whole cohort of patients (*n* = 107)

#### 3.1.1 Patient characteristics

Overall, 107 patients were analysed from 11 haemato‐oncology centres: 64 patients with de novo DLBCL, 18 patients with transformed DLBCL, 13 patients with high‐grade LBCL and 12 patients with primary mediastinal LBCL (PMBCL). These patients were treated at five centres certified for CAR‐Tx. Tisa‐cel was administered to 77 patients (72%), and axi‐cel was administered to 30 patients (28%). Their basic characteristics are summarised in Table 1.

**TABLE 1:** Baseline comparison of patients in durable remission and those with CAR T‐cell failure.

Characteristic	Durable complete remission (*n* = 40 (%)	Relapse/progression/death after CAR T‐cell therapy *n* = 67 (%)	*p* Value
Median age at apheresis (range), years	56 (23–73)	63 (21–77)	0.010
Subtype of lymphoma			
DLBCL	24 (60)	40 (60)	0.006
HGBCL	1 (3)	12 (18)	
PMBCL	9 (23)	3 (5)	
tDLBCL	6 (15)	12 (18)	
Type of product: tisa‐cel	23 (58)	54 (81)	0.014
Haemoglobin < LNR before lymphodepletion	29 (73)	58 (89)	0.035
Missing	0 (0)	2 (3)	
Albumin <LNR (35 g/L) before lymphodepletion	3 (8)	18 (27)	0.013
Best overall response rate after CAR T			
CR	40 (100)	14 (21)	<0.001
PR	0 (0)	10 (15)	
SD	0 (0)	8 (12)	
PD	0 (0)	35 (52)	

Abbreviations: CR, complete remission; DLBCL, diffuse large B‐cell lymphoma; HGBCL, high grade B‐cell lymphoma; LNR, limit of normal range; n, number; PD, progressive disease; PMBCL, primary mediastinal B‐cell lymphoma; PR, partial remission; SD, stable disease; tDLBCL, transformed DLBCL.

#### 3.1.2 Treatment response and clinical outcome after CAR T‐cell therapy

**FIGURE 1:**
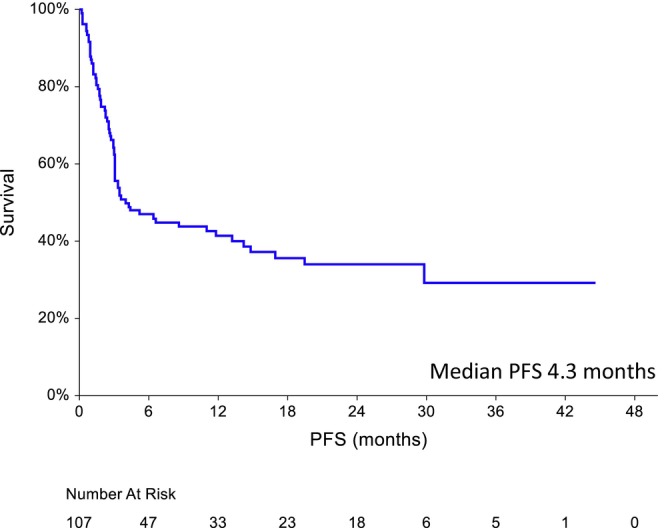
Progression‐free survival (*n* = 107).

The best ORR and CR rate were 60% (*n* = 64) and 50% (*n* = 53), respectively. The median follow‐up was 17.8 months (range 2.1–44.6) for surviving patients. Among the eleven patients who achieved the best response to PR after CAR‐Tx, 10 progressed. Fifty patients died: 45 due to progressive disease (PD), three due to infectious complications, one from other causes and one from unknown causes. The median PFS (Figure 1) and OS were 4.3 months (95% CI 3.1–11.8) and 26.4 months (95% CI 9.5–29.8), respectively, in all patients. The estimated 6‐month PFS and OS were 47% and 66%, respectively, and the estimated 12‐month PFS and OS were 41% and 56%, respectively. The results of univariate and multivariate Cox regression analyses for PFS and OS are shown in Table 2. In contrast to ‘bulky disease’ (≥5 cm), the response to bridging therapy lost its predictive value in multivariate analysis for OS/PFS, probably due to the small number of patients. The histological type and poor ECOG PS were not predictive of OS in the multivariate analysis, while an albumin concentration lower than the limit of the normal range (LNR) before LD was. The best response to CAR‐Tx and no ‘bulky disease’ predicted both PFS and OS.

**TABLE 2:** Univariate analysis and multivariate analysis for overall survival (OS) and progression‐free survival (PFS) (*n* = 107).

Variable	Overall survival (*n* = 107)			Progression‐free survival (*n* = 107)		
	Hazard ratio	95% CI	*p* Value	Hazard ratio	95% CI	*p* Value
Univariate analysis Kaplan*–*Meier method						
Diagnosis ‘other BCL’ vs. PMBCL	2.51	1.13–5.61	0.104	3.97	2.06–7.67	0.011
LDH (μkat/L) at apheresis	2.17	1.23–3.84	0.019	1.80	1.10–2.94	0.029
Response SD/PD before CAR T‐cell therapy	2.24	1.27–3.92	0.012	1.66	1.02–2.71	0.055
Lymphodepletion consisting of bendamustine	2.12	0.93–4.84	0.020	1.87	0.88–3.98	0.038
Type of product—tisa‐cel	1.52	0.82–2.82	0.232	1.88	1.12–3.16	0.036
Response SD/PD to CAR T‐cell therapy	6.62	3.52–12.44	<0.0001	8.17	4.38–15.24	<0.0001
C‐reactive protein >LNR (mg/L) before lymphodepletion	1.91	1.09–3.35	0.027	1.37	0.85–2.22	0.205
LDH > LNR (μkat/L) before lymphodepletion	2.43	1.39–4.26	0.005	1.76	1.09–2.84	0.029
EN involvement before lymphodepletion	2.41	1.38–4.19	0.003	1.94	1.20–3.13	0.007
ECOG PS > 1 before lymphodepletion	2.59	1.26–5.33	0.001	2.29	1.20–4.36	0.001
‘Bulky disease’ ≥5 cm before lymphodepletion	2.15	1.20–3.83	0.006	1.58	0.96–2.62	0.055
Albumin < LNR before lymphodepletion	2.86	1.29–6.33	0.00030	2.21	1.11–4.38	0.003
Expansion of CAR T‐cell <20 cells/μL	1.86	0.97–3.59	0.0403	1.69	(0.94–3.03)	0.052
Multivariate analysis						
Diagnosis: other BCL vs. PMBCL	—	—	NS	3.52	1.03–11.95	0.020
ECOG PS > 1 before lymphodepletion	—	—	NS	1.88	1.09–3.22	0.028
‘Bulky disease’ ≥5 cm before lymphodepletion	2.34	1.28–4.29	0.006	2.32	1.37–3.93	0.002
Albumin < LNR before lymphodepletion	2.04	1.10–3.81	0.030	—	—	NS
Response SD/PD response to CAR T‐cell therapy	8.48	4.31–16.71	<0.0001	18.68	9.14–38.18	<0.0001
	6‐month and 12‐month OS 66% (95% CI 56.5–74.8) 56% (95% CI 45.7–65.4)			6‐month and 12‐month PFS 47% (95% CI 37.4–56.5), 41% (95% CI 31.7–50.9)		

Abbreviations: CAR, chimeric antigen receptor; CAR‐T, chimeric antigen receptor; CI, confidence interval; ECOG PS, Eastern Cooperative Oncology Group Performance Status; EN, extranodal; LDH, lactate dehydrogenase; LDH, lactate dehydrogenase; LNR, limit of normal; n, number.; PD, progressive disease; PMBCL, primary mediastinal B‐cell lymphoma; SD, stable disease; SD/PD, stable disease/progression.

#### 3.1.3 Failure after CAR‐Tx

At the time of analysis, 63 patients had failed CAR‐Tx. The differences in characteristics before or at the time of CAR‐Tx between patients with long‐term durable CR and patients with R/P DLBCL are summarised in Table 1. Patients at the time of APH were older than patients without CAR T‐cell failure, and their haemoglobin and albumin levels before LD were more frequently below the respective LNRs. Repeat tumour biopsy was performed in 18 patients (29%) after CAR T‐cell failure, and 12 patients (66%) were positive for CD19 antigen. Otherwise, all the R/P histologies were the same as those of the primary samples. The characteristics of the R/P patients after CAR‐Tx are summarised in Table 3. Most patients with a diagnosis other than PMBCL progressed after CAR‐Tx, while only three of the 12 patients with PMBCL progressed. Fourteen patients progressed early (<1 month), 24 patients progressed within 2–3 months and 25 patients progressed >3 months after CAR‐Tx. The median follow‐up for patients with CAR T‐cell survival failure at R/P after CAR‐Tx was 10.6 months (range 2.4–23.2). The majority of the R/P patients had advanced‐stage disease, elevated LDH, and EN involvement, and more than half of the patients had ‘bulky disease’ ≥5 cm and an ECOG PS >1 at progression. The persistence of A subset of the CAR T‐cells was tested in a subset of the patients (*n* = 30), and CAR‐T cells were present in more than half of the patients (*n* = 57%) (Table 3).

**TABLE 3:** Baseline characteristics of the patients at relapse/progression after CAR T‐cell therapy (*n* = 63).

Variable	Number of patients (n, %)
Subtype of lymphoma	
DLBCL	38 (60)
HGBCL	10 (16)
PMBCL	3 (5)
tDLBCL	12 (19)
Age (median, range)	63 (22–78)
Female	22 (35)
Peak expansion after CAR T‐cell infusion (cells/μL) (median, range)	54.5 (0–989)
Missing	11
Peak expansion—day after CAR T‐cell infusion (cells/μL) (median, range)	11 (1–120)
‘Bulky disease’ ≥5 cm	37 (59)
CNS involvement	0 (0)
ECOG PS 0–1	33 (52)
LDH > LNR(μkat/L)	52 (83)
EN involvement	50 (79)
Type of product	
Tisa‐cel	12 (19)
Axi‐cel	51 (81)
Tumour biopsy—yes	18 (29)
CD19 positivity—yes	12 (66)
CD19 positivity unknown	6 (34)
Persistence of CAR T‐cells	
Yes	17 (57)
No	13 (43)
Missing	33 (52)
Ann Arbor stage III–IV	46 (73)
Albumin < LNR (35 g/L)	
Yes	16 (25)
No	39 (62)
Missing	8 (13)
IPI (0–1/2/3/4‐5/NA) (*n* = 36)	3/4/7/20/1
AA IPI 0/1/2/3 (*n* = 27)	3/2/12/10

Abbreviations: AA IPI age‐adjusted IPI; CAR, chimeric antigen receptor; CNS, central nervous system; DLBCL, diffuse large B, cell lymphoma; ECOG PS, Eastern Cooperative Oncology Group Performance Status; EN, extranodal; HGBCL, high grade B‐cell lymphoma; IPI, International Prognostic Index; LDH, lactate dehydrogenase; LNR, limit of normal range; n, number; NA, not available; PMBCL, primary mediastinal B, cell lymphoma; tDLBCL, transformed DLBCL.

Of the 63 patients, 39 (62%) received further therapy, and 24 (38%) did not. The latter received only supportive and palliative care, mostly due to the rapidity of their progression and their poor PS. Twenty‐one (87.5%) patients who did not receive further treatment had stable disease or progression as their best response after CAR‐Tx, compared to 22 (56.4%) of those who received further therapy. The differences between these groups are listed in Table 4. In multivariate analysis, four risk factors for lack of further treatment were identified, three of which were present before or at the time of CAR‐Tx: elevated LDH before APH, EN involvement and elevated ferritin before LD. The last risk factor was the ECOG PS at R/P status after CAR T‐cell failure (Table 4).

**TABLE 4:** Baseline characteristics of the analysed patients who experienced relapse/progression after CAR T‐cell therapy (*n* = 63).

Characteristics	Treatment after CART‐cell failure (GK excluded), *n* = 39 (%)	Without salvage treatment after CAR T‐cell failure, n = 24 (%)	*p* Value
‘Bulky disease’ ≥5 cm before lymhodepletion	14 (35.9)	17 (70.8)	0.009
ECOG PS 0–1 before lymphodepletion	31 (79.5)	11 (45.8)	0.012
EN involvement before lymphodepletion	20 (51.3)	22 (91.7)	0.001
LDH > LNR at apheresis	25 (64.1)	22 (91.7)	0.018
IPI before lymphodepletion	*N* = 21	*N* = 15	
1	5 (23.8)	1 (6.7)	
2	9 (42.9)	0 (0)	0.002
3	4 (19)	5 (33.3)	
4–5	3 (14.3)	9 (60)	
AA IPI before lymphodepletion	*N* = 18	*N* = 9	
0	3 (16.7)	0 (0)	
1	6 (33.3)	0 (0)	0.035
2	6 (33.3)	3 (33.3)	
3	3 (16.7)	6 (66.7)	
Ferritin before lymhodepletion > LNR (μg/L)	24 (63.2)	22 (95.7)	0.005
Missing	1	1	
C‐reactive protein > LNR (mg/L)	20 (51.3)	19 (82.6)	0.016
Missing	0	1	
Albumin < LNR (35 g/L) before lymphodepletion	6 (15.4)	12 (52.2)	0.004
Missing		1	
Best overall response rate after CAR T			
CR/PR	17 (43.6)	3 (12.5)	0.012
SD/PD	22 (56.4)	21 (87.5)	
Presence of EN at R/P disease	26 (66.7)	24 (100)	0.001
Presence of ‘bulky disease’ ≥ 5 cm at R/P disease	17 (43.6)	20 (83.3)	0.003
Albumin <LNR (35 g/L) at R/P disease	6 (18.2)	10 (45.5)	0.038
Missing	6	2	
ECOG PS 0–1at R/P disease	27 (69.2)	5 (20.8)	0.000
Ann Arbor stage I–II at R/P disease	15 (38.5)	2 (8.3)	0.009

Multivariate analysis‐logistic regression Risk factors for no treatment at R/P after CAR T‐cell failure	Odds ratio	95% CI	***p* Value**
Variable			
LDH > LNR (μkat/L) at apheresis	9.59	1.26–73.02	0.016
EN involvement before lymphodepletion	19.33	2.92–127.81	0.000
Ferritin before lymhodepletion > LNR (μg/L)	13.87	1.19–160.43	0.015
ECOG PS > 1 at R/P disease	6.49	1.34–31.36	0.013

Abbreviations: AA IPI, age‐adjusted IPI;CAR, chimeric antigen receptor; CR, complete remission; ECOG PS, Eastern Cooperative Oncology Group Performance Status; EN, extranodal; IPI, International Prognostic Index; LDH, lactate dehydrogenase; LNR, limit of normal range; n, number; PD, progressive disease; PR, partial remission; R/P, relapse/progression; SD, stable disease.

#### 3.1.4 Treatment, response to treatment and clinical outcome after CAR T‐cell failure

**FIGURE 2:**
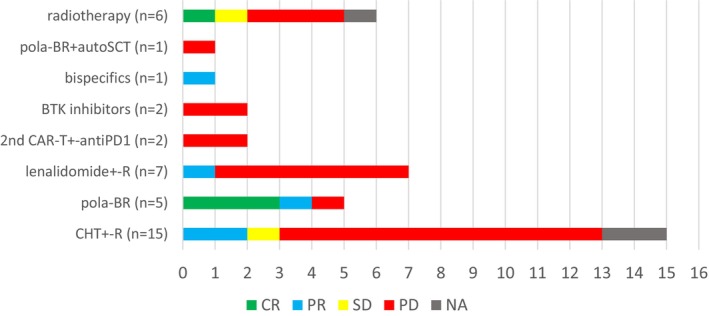
Treatment modalities and response after first‐line postCAR T‐cell therapy. BTK, Bruton kinase; CAR, chimeric antigen receptor; CHT, chemotherapy; CR, complete remission; NA, not available; pola‐BR, polatuzumab vedotin‐bendamustine + rituximab; PD1, programmed death 1; PD, progressive disease; PR, partial remission; R, rituximab; SD, stable disease.

As the 1st‐line treatment after R/P after CAR‐Tx, six patients underwent radiotherapy (RT) and 33 patients underwent systemic therapy (excluding glucocorticoids): lenalidomide ± rituximab in seven patients, polatuzumab + bendamustine and rituximab (pola‐BR) in six patients, chemotherapy ± rituximab in 15 patients, bispecific antibody in one patient, CAR T‐cell reinfusion ± anti‐PD1 treatment in two patients and therapy with Bruton's tyrosine kinase inhibitors in two patients. The ORR was 22% (CR 8%). The response rates after different 1st‐line regimens are shown in Figure 2. Sixteen patients were further treated with 2nd‐line therapy (RT in two patients, systemic therapy in 13 patients and the same second CAR T‐cell product in one patient). A response was achieved in four patients (25%), one whom achieved CR. Six patients received 3rd‐line therapy, one of whom received 4th‐line of therapy. Bispecific antibodies (BsAbs) at any timepoint of treatment after R/P disease were indicated in six patients. In five patients, BsAbs were administered as 2nd or next‐line therapy in three patients (CR in one patient, PR in two patients). One patient received allogeneic SCT as a 3rd‐line treatment and achieved CR but died from infection. A second infusion of the same CAR T‐cell product after 1st‐line treatment (tisa‐cel; one patient) did not achieve any transient response.

**FIGURE 3:**
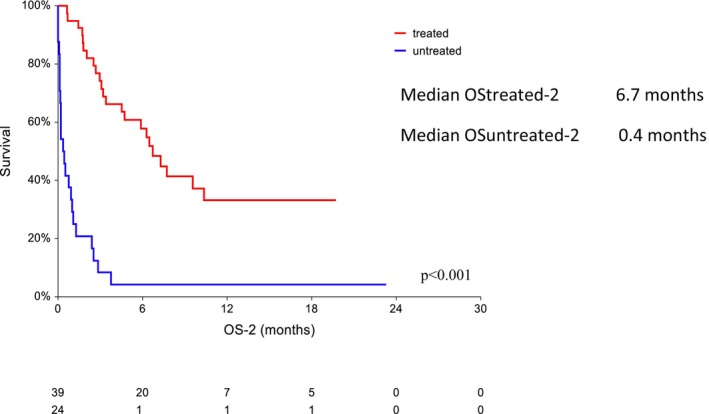
Overall survival (OS‐2; *n* = 63)—treated vs. untreated patients.

Among the 63 patients who experienced CAR T‐cell failure, 46 (73%) died. The median OS‐2 for patients whose CAR‐Tx failed (*n* = 63) was 3 months (95% CI 1.8–4.7) from the time of diagnosis of R/P lymphoma (6‐month and 12‐month OS‐2 37.3% [95% CI 25.2–49.4] and 22.3% [95% CI 11–33.7], respectively). The median OS‐2 was 0.2 months for patients who progressed within 1 month after CAR T‐cell therapy (95% CI 0.1–3.8) and 3.2 months for patients who progressed later (95% CI 2.4–6.7). Long‐term survival was not achieved in any of these patients with early R/P. The median OS‐2 in treated patients was 6.7 months (6‐month and 12‐month OS‐2: 57.9% [95% CI 42.1–73.7] and 33.1% [95% CI 16.3–49.9], respectively). For untreated patients, the median OS‐2 was only 0.4 months and the median 6‐month OS‐2 was only 4.2% [95% CI 0–12.2] (Figure 3). The median PFS‐2 in treated patients was 3.2 months (95% CI 2.3–4), and the 6‐month and 12‐month PFS‐2 in these patients were 28.5% (95% CI 13.8–43.2) and 21.4% (95% CI 7.4–35.3), respectively.

#### 3.1.5 Univariate analysis and multivariate Cox regression analysis for OS‐2 in all patients who failed to respond to CAR‐Tx (*n* = 63)

##### Univariate analysis

Several risk factors for a shorter OS‐2 (*n* = 63) were already present before CAR‐Tx and were still there at R/P: LDH > LNR (*p* = 0.004, *p* = 0.013), EN involvement (*p* = 0.011, *p* = 0.001), PS ECOG >1 (*p* = 0.007, *p* < 0.0001), ‘bulky disease’ ≥5 cm (*p* = 0.0006, *p* < 0.0001), albumin < LNR (*p* = 0.010, *p* < 0.0001 and refractory status before CAR‐Tx (*p* = 0.009, *p* = 0.039)). However, elevated ferritin and CRP were significant only before LD (*p* = 0.050 and *p* = 0.0004, respectively). In addition, the time to progression after CAR‐Tx (>1 month vs. ≤1 month) (*p* = 0.005) was a significant predictor of OS‐2.

##### Multivariate analysis

According to the multivariate analysis, only one factor present before LD predicted shorter OS‐2: CRP > LNR, *p* = 0.0001. The five factors associated with progression were no treatment response (SD/PD) to CAR‐Tx (*p* = 0.009), albumin < LNR (*p* < 0.0001), ECOG PS > 1 (*p* < 0.0001), EN involvement (*p* = 0.0007) and stage III‐IV (*p* = 0.044).

##### Univariate analysis and multivariate analysis for OS treated‐2 and PFS treated‐2 in patients who received therapy (*n* = 39) after CAR‐Tx failure

**FIGURE 4:**
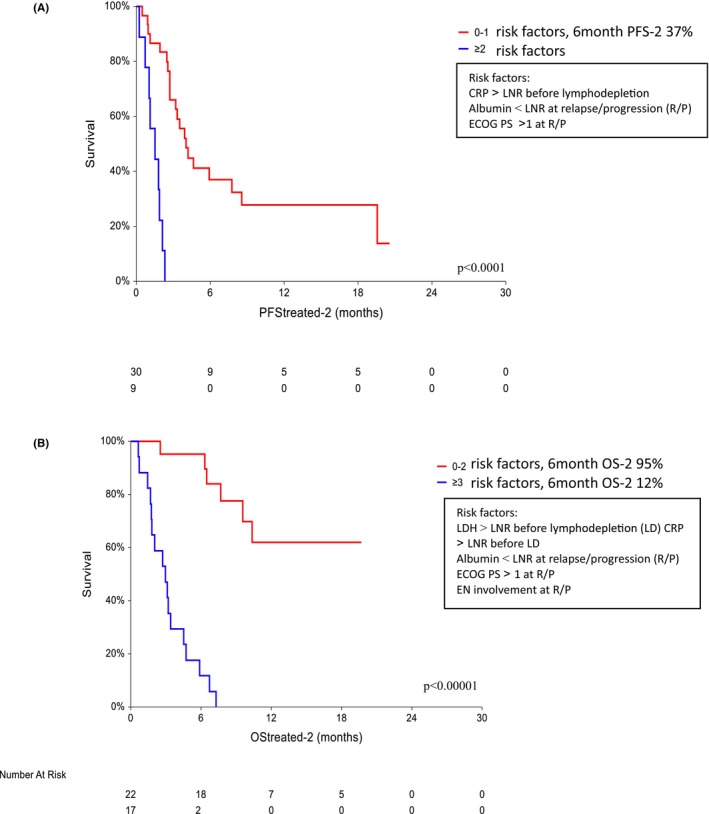
Progression‐free survival (PFS treated‐2;A) and overall survival (OS treated‐2; B) of patients who failed to respond to CAR T‐cell therapy (*n* = 39).

The risk factors for PFS treated‐2 and OS treated‐2 according to univariate analysis and multivariate analysis are shown in Table 5. The risk factors for a shorter PFS treated‐2 and OS treated‐2 again included several factors presented before LD and at R/P. All factors predictive of PFS treated‐2 were also predictive of OS treated‐2. In addition, LDH > LNR before LD and EN involvement at R/P predicted the OS treated‐2 duration (Table 5 **)**. The presence of ≥2 factors compared to the presence of 0–1 factors was associated with inferior PFS treated‐2 (*p* < 0.00001, HR 5.39, 95% CI 1.37–21.22), ≤ 1 factor (median 4 months), and ≥2 factors (median 1.5 months); a 6‐month PFS treated‐2 was achieved in 37% of those with 0–1 factors. The presence of ≥3 risk factors worsened OS treated‐2 (*p* < 0.00001, HR 9.26, 95% CI 3.53–24.3; a 6‐month OS treated‐2 was achieved in 95% of those with 0–2 factors vs. 12% of those with ≥3 factors; median not reached vs. median = 3 months) (Figure 4). According to multivariate analysis, the risk factors for OS‐2 and OS treated‐2 differed. Only one factor, elevated CRP, which was present before LD, predicted OS‐2 and elevated LDH before LD predicted OS treated‐2. While five factors present at progression were predictive of OS‐2 (SD/PD after CAR‐Tx, ECOG PS > 1, decreased albumin, stage III‐IV, EN involvement), only three (ECOG PS, EN involvement, albumin) predicted OS treated‐2. For PFS treated‐2, only CRP before LD together with ECOG PS > 1 and decreased albumin after CAR T‐cell failure were predictive.

**TABLE 5:** Univariate and multivariate analyses of patients treated after CAR T‐cell failure: Overall survival (OS treated‐2, *n* = 39) and progression‐free survival (PFS treated‐2, *n* = 39).

Variable	Hazard ratio	95% CI	Overall survival in treated patients (OS treated‐2), *n* = 39	Hazard ratio	95% CI	Progression‐free survival in treated patients (PFS treated‐2), *n* = 39
			*p* Value			*p* Value
Univariate analysis Kaplan*–*Meier method						
LDH > LNR (μkat/L) before lymphodepletion	3.44	1.52–7.80	0.008	1.22	0.59–2.53	0.589
‘Bulky disease’ ≥ 5 cm before lymphodepletion	2.11	0.84–5.33	0.059	1.63	0.72–3.68	0.170
C‐reactive protein > LNR (mg/L) before lymphodepletion	2.43	1.05–5.61	0.031	2.45	1.15–5.18	0.009
SD/PD before CAR T‐cell infusion	3.20	1.41–7.26	0.012	1.70	0.83–3.49	0.147
‘Bulky disease’ ≥5 cm at R/P disease	3.37	1.39–8.12	0.002	2.56	1.18–5.56	0.005
Albumin < LNR at R/P disease	3.77	0.88–16.19	0.002	3.37	0.82–13.74	0.004
ECOG PS > 1 at R/P disease	2.79	1.04–7.55	0.009	2.53	1.05–6.11	0.008
LDH > LNR (μkat/L) at R/P disease	4.28	1.74–10.55	0.031	4.26	1.98–9.17	0.008
EN involvement at R/P disease	4.11	1.81–9.32	0.004	2.27	1.10–4.65	0.033
Ann Arbor stage III–IV	3.22	1.42–7.30	0.014	2.87	1.40–5.87	0.005
Multivariate analysis						
LDH > LNR (μkat/L) before lymphodepletion	7.03	1.67–29.64	0.002	—	—	NS
C‐reactive protein > LNR (mg/L) before lymphodepletion	6.84	2.13–22	0.0004	11.20	3.76–33.37	<0.0001
Albumin < LNR at R/P disease	11.99	3.02–47.69	0.0004	12.11	3.55–41.38	<0.0001
ECOG PS > 1 at R/P disease	8.48	2.5–28.75	0.0003	3.69	1.49–9.11	0.005
EN involvement at R/P disease	4.52	1.08–18.99	0.023	—	—	NS

Abbreviations: CAR, chimeric antigen receptor; CI, confidence interval; ECOG PS, Eastern Cooperative Oncology Group Performance Status; EN, extranodal; LDH, lactate dehydrogenase; LNR, limit of normal range; NS, not significant; OS treated‐2, time from R/P diagnosis after CAR T‐cell failure to death in treated patients; PFS treated‐2, time from R/P diagnosis to next progression/relapse or death in treated patients after CAR T‐cell failure; R/P, relapse/progression; SD/PD, stable disease/progression.

## 4 DISCUSSION

To our knowledge, this is the first and largest real‐world analysis of CAR T‐cell‐treated LBCL patients from Central and Eastern European countries. We retrospectively analysed 107 consecutive patients from the Czech Republic and Slovakia treated with tisa‐cel or axi‐cel between 2019 and 2022, with a special focus on patients whose CAR‐Tx failed. Patients characteristics before APH and at LD were comparable to those of registration studies, as well as to other real‐world data.^11^, ^12^, ^21^, ^47^, ^48^, ^49^, ^50^ The overall response, CR rates and median PFS and OS were also within the expected ranges.^11^, ^47^, ^48^

We compared the outcomes of our patients after CAR‐Tx failure to those of patients in previously published studies. In their analysis of 238 patients, di Blasi et al.^51^ reported a median OS‐2 of 5.2 months and a PFS‐2 of 2.8 months for all failed patients, 65% of whom received further treatment. In two 2023 studies, Zurko et al.^52^ reported a median PFS treated‐2 of 2.8 months and a median OS‐2 5.5 months, while Alacron et al.^53^ reported a median EFS‐2 of 1.9 months and a median OS treated‐2 of 8.5 months. In agreement with our study, patients who progressed within 1 month after CAR‐Tx had particularly poor outcomes.^51^, ^52^, ^53^, ^54^, ^55^, ^56^ We chose slightly different methods of data reporting, stressing the importance of both OS‐2 and OS treated‐2, and we reported PFS after CAR‐Tx failure only for patients who received further treatment (PFS treated‐2). We believe this makes sense, as it is sometimes difficult to determine the date of progression in untreated patients. In contrast to our study, other authors also did not mention the risk for not receiving treatment. Among our patients who received further treatment after CAR T‐cell failure, the response rates to the 1st‐line treatment were low. With the exclusion of regimen polatuzumab‐bendamustine and rituximab (pola‐BR), which was analysed separately, most patients were treated with immunochemotherapy (*n* = 15, ORR 15%, CR 0%). These results are worse than those of Zurko et al.^52^, ^56^ (*n* = 17, ORR 35%, CR 12%) but better than those of di Blasi et al.^51^ (*n* = 31, ORR 8%, CR 4%). As in our study, no CR after immunochemotherapy was observed by Alarcon et al. (*n* = 15 patients).^53^ The finding that conventional immunochemotherapy is an ineffective treatment after CAR‐Tx failure was also confirmed by Nastoupil et al.^55^ in 2023 (*n* = 43 patients, ORR 14%, CR 7%). Pola‐BR, on the contrary, was an effective salvage regimen in our cohort, where five of six patients responded (with CR in four patients). One patient who was consolidated with autologous stem cell transplantation (autoSCT) progressed early after transplantation. Only one of four patients received bispecific antibodies (BsAbs) as a first‐line treatment after CAR‐Tx failure. That one patient achieved PR, while three of five patients receiving BsAbs as a next‐line treatment responded (one achieved CR, two achieved PR).

Factors predicting the ability to receive treatment after R/P or survival in our cohort may be divided into patient‐related factors (ECOG PS, inflammatory status) and disease‐related factors (extent of the disease, LDH [indirectly tumour burden], tumour intrinsic factors).

Most of the factors predicting the probability of further treatment according to multivariate analysis were already present at APH and before LD and consisted of two disease‐related factors (EN involvement and LDH) and one inflammatory factor (ferritin). The ECOG PS at R/P was an additional prognostic factor. As the probability of receiving further treatment at R/P affects further survival, patients with the abovementioned pretreatment characteristics deserve special attention. Factors for not receiving further treatment in R/P patients were mostly different from factors predicting OS treated‐2 or PFS treated‐2 in failed patients who received further treatment, but again, two factors measured before treatment (CRP and LDH before LD) predicted OS treated‐2 and CRP before LD for PFS treated‐2 as well. Again, simple measurement of these two laboratory markers may draw closer attention to subsets of ultrahigh‐risk patients. Three other factors for the survival of treated patients who failed to respond (low albumin concentration, ECOG PS and EN involvement) were present at R/P. Even if we have early knowledge of parameters influencing outcomes, it is difficult to address them properly. Currently, we are unable to address parameters of inflammation, so we must focus on improving disease‐related factors and poor performance status. ‘Bulky disease’ (≥5 cm, predictive of survival), LDH and EN involvement (predictive for not receiving treatment after CAR‐Tx R/P) may be addressed by bridging therapy. ‘Lymphoma debulking’ before CAR‐Tx has a confirmed role,^21^, ^26^, ^27^, ^41^, ^57^, ^58^ and it may also improve performance status, but too aggressive bridging may in fact worsen this parameter and negatively impact survival outcomes.

Bispecific antibodies are direct CAR T‐cell competitors in R/R DLBCL patients and are offered as a possibly definitive treatment.^59^, ^60^, ^61^, ^62^, ^63^ Bispecifics targeting different antigens are effective after CAR T‐cell failure, and in a retrospective analysis using a matched control cohort from the French registry, prior treatment with BsAbs did not reduce the effect of CAR‐Tx.^64^, ^65^ These therapies may be successful, not only when overt progression occurs. It would be of interest whether a short course of BsAbs alone or in combination with chemotherapy might be used for ‘bridging’ or whether patients achieving only partial remission on BsAbs monotherapy might be successfully consolidated by CAR‐Tx. Another tool that may prevent deterioration of ECOG PS and progression of disease before CAR‐Tx is shortening CAR T‐cell production.^66^

Closer follow‐up after treatment should also be employed. Currently, the first PET/CT scan is recommended as early as 1 month after CAR T‐cell infusion, but the possibility of an even earlier assessment of response needs to be examined. The inclusion of minimal residual disease monitoring (MRD) by noninvasive circulating tumour (ct) DNA analysis could improve prognostication beyond PET/CT. In two studies, ctDNA assessments were used to predict the outcome of patients receiving axi‐cel or tisa‐cel for LBCL therapy.^67^, ^68^

Lenalidomide maintenance was used with some success in patients with early evidence of treatment failure or in only partial remission after CAR‐Tx. Patients who achieve a PR after CAR‐Tx should be seriously considered for next‐line treatment and possibly for allogeneic SCT. By any means, BsAbs are the most promising group of agents for the treatment of patients with CAR T‐cell failure.^51^, ^52^, ^56^, ^60^, ^61^, ^62^, ^63^, ^69^, ^70^, ^71^, ^72^

Despite its multicentre nature, our study has several limitations. First, this was a retrospective analysis. Second, we treated the patients with two different CAR T‐cell products. Third, the group of failing patients was relatively small, and their choice of individual therapy options might be biased by patient and physician preferences, as well as by reimbursement issues.

In summary, our retrospective study confirmed the unfavourable prognosis of patients with LBCL who progressed or relapsed after treatment with the commercially available CAR T‐cell products tisa‐cel and axi‐cel. We confirmed the limited role of salvage immunochemotherapy, with the possible exception of the pola‐BR regimen. BsAbs were relatively effective even when used in later lines of treatment after CAR‐Tx failure. We identified prognostic factors for progression after CAR‐Tx, for the ability to receive further treatment, and for outcomes after progression in untreated and treated patients. We found that several factors predictive of the R/P ratio after CAR‐Tx, the probability of further treatment, and the outcome after relapse were present even before the patients started CAR T‐cell infusion. These findings can form a basis for further refinement of the management of CAR T‐cell therapy, enabling the identification of an ultrahigh‐risk subgroup of CAR T‐cell candidates who may benefit from aggressive pre‐CAR T‐cell therapy (‘debulking’). Early evaluation of the response after CAR‐Tx is needed to address CAR‐Tx failure (early MRD monitoring, early restaging [CT/PET] after CAR‐Tx). When only a PR is achieved after CAR‐Tx, early intervention is appropriate due to the high risk of CAR‐Tx failure.

## 5 CONCLUSION

To prevent R/P disease after CAR‐Tx and reduce the number of patients who cannot receive treatment after eventual R/P, we need to address the ECOG PS and systemic inflammatory status of patients during the indication process and consider appropriate lymphoma debulking strategies before CAR‐Tx. To give more relapsing patients the opportunity for further treatment, early evaluation of the treatment response is necessary, ideally with the inclusion of ctDNA‐MRD monitoring when it becomes available. The incorporation of BsAbs into bridging therapy or early after R/P should be investigated. Finally, better understanding of CAR T‐cell resistance and the development of new CAR T‐cell products and strategies will increase the efficacy of this treatment modality and hopefully reduce the number of failed LBCL patients in the future.

## AUTHOR CONTRIBUTIONS

**Alice Sýkorová:** Conceptualization (lead); formal analysis (equal); investigation (supporting); methodology (lead); writing – original draft (lead); writing – review and editing (lead). **František Folber:** Conceptualization (supporting); investigation (equal); methodology (supporting); validation (supporting). **Kamila Polgárová:** Investigation (equal); validation (supporting). **Heidi Móciková:** Investigation (supporting); validation (equal). **Juraj Ďuraš:** Investigation (supporting); validation (supporting). **Kateřina Steinerová:** Investigation (supporting); validation (equal). **Aleš Obr:** Investigation (supporting); validation (supporting). **Adriana Heindorfer:** Investigation (supporting). **Miriam Ladická:** Investigation (supporting). **Ľubica Lukáčová:** Investigation (supporting). **Erika Čellárová:** Investigation (supporting). **Ivana Plameňová:** Investigation (supporting). **David Belada:** Investigation (supporting); validation (supporting). **Andrea Janíková:** Validation (supporting). **Marek Trněný:** Conceptualization (supporting); methodology (supporting); validation (supporting); writing – review and editing (supporting). **Tereza Jančárková:** Investigation (supporting). **Vít Procházka:** Investigation (supporting); methodology (supporting); validation (supporting). **Andrej Vranovský:** Investigation (supporting). **Margaréta Králiková:** Investigation (supporting). **Jan Vydra:** Investigation (supporting); validation (equal). **Lukáš Smolej:** Methodology (supporting). **Ľuboš Drgoňa:** Investigation (supporting). **Martin Sedmina:** Investigation (supporting). **Eva Čermáková:** Methodology (supporting). **Robert Pytlík:** Conceptualization (equal); investigation (equal); methodology (equal); validation (lead); writing – original draft (equal); writing – review and editing (equal).

## FUNDING INFORMATION

This work was supported by the Ministry of Health, Czech Republic under grant MH CZ‐DRO (UHHK, 00179906), under grant AZV NU21‐03‐00411 and by Charles University in Prague under the Cooperatio program, research area “Oncology and Haematology”.

## Data Availability

Data sharing is not applicable to this article as no new data were created or analyzed in this study.

## References

[1] Crump M , Kuruvilla J , Couban S , et al. Randomized comparison of gemcitabine, dexamethasone, and cisplatin versus dexamethasone, cytarabine, and cisplatin chemotherapy before autologous stem‐cell transplantation for relapsed and refractory aggressive lymphomas: NCIC‐CTG LY.12. J Clin Oncol. 2014;32(31):3490‐3496. 25267740 10.1200/JCO.2013.53.9593

[2] Van Den Neste E , Schmitz N , Mounier N , et al. Outcome of patients with relapsed diffuse large B‐cell lymphoma who fail second‐line salvage regimens in the international CORAL study. Bone Marrow Transplant. 2016;51(1):51‐57. 26367239 10.1038/bmt.2015.213

[3] Hamadani M , Hari PN , Zhang Y , et al. Early failure of frontline rituximab‐containing chemo‐immunotherapy in diffuse large B cell lymphoma does not predict futility of autologous hematopoietic cell transplantation. Biol Blood Marrow Transplant. 2014;20(11):1729‐1736. 25008330 10.1016/j.bbmt.2014.06.036 PMC4194275

[4] Zhang Y , Zhang Z . The history and advances in cancer immunotherapy: understanding the characteristics of tumor‐infiltrating immune cells and their therapeutic implications. Cell Mol Immunol. 2020;17(8):807‐821. 32612154 10.1038/s41423-020-0488-6 PMC7395159

[5] Hwu P , Yang JC , Cowherd R , et al. In vivo antitumor activity of T cells redirected with chimeric antibody/T‐cell receptor genes. Cancer Res. 1995;55(15):3369‐3373. 7614473

[6] Kershaw MH , Westwood JA , Parker LL , et al. A phase I study on adoptive immunotherapy using gene‐modified T cells for ovarian cancer. Clin Cancer Res. 2006;12(20 Pt 1):6106‐6115. 17062687 10.1158/1078-0432.CCR-06-1183 PMC2154351

[7] Gross G , Waks T , Eshhar Z . Expression of immunoglobulin‐T‐cell receptor chimeric molecules as functional receptors with antibody‐type specificity. Proc Natl Acad Sci USA. 1989;86(24):10024‐10028. 2513569 10.1073/pnas.86.24.10024 PMC298636

[8] Imai C , Mihara K , Andreansky M , et al. Chimeric receptors with 4‐1BB signaling capacity provoke potent cytotoxicity against acute lymphoblastic leukemia. Leukemia. 2004;18(4):676‐684. 14961035 10.1038/sj.leu.2403302

[9] Krause A , Guo HF , Latouche JB , Tan C , Cheung NK , Sadelain M . Antigen‐dependent CD28 signaling selectively enhances survival and proliferation in genetically modified activated human primary T lymphocytes. J Exp Med. 1998;188(4):619‐626. 9705944 10.1084/jem.188.4.619 PMC2213361

[10] Kalos M , Levine BL , Porter DL , et al. T cells with chimeric antigen receptors have potent antitumor effects and can establish memory in patients with advanced leukemia. Sci Transl Med. 2011;3(95):95ra73. 10.1126/scitranslmed.3002842 PMC3393096 21832238

[11] Schuster SJ , Bishop MR , Tam CS , et al. Tisagenlecleucel in adult relapsed or refractory diffuse large B‐cell lymphoma. N Engl J Med. 2019;380(1):45‐56. 30501490 10.1056/NEJMoa1804980

[12] Neelapu SS , Locke FL , Bartlett NL , et al. Axicabtagene Ciloleucel CAR T‐cell therapy in refractory large B‐cell lymphoma. N Engl J Med. 2017;377(26):2531‐2544. 29226797 10.1056/NEJMoa1707447 PMC5882485

[13] Abramson JS , Palomba ML , Gordon LI , et al. Lisocabtagene maraleucel for patients with relapsed or refractory large B‐cell lymphomas (TRANSCEND NHL 001): a multicentre seamless design study. Lancet Lond Engl. 2020;396(10254):839‐852. 10.1016/S0140-6736(20)31366-0 32888407

[14] Fowler NH , Dickinson M , Dreyling M , et al. Tisagenlecleucel in adult relapsed or refractory follicular lymphoma: the phase 2 ELARA trial. Nat Med. 2022;28(2):325‐332. 34921238 10.1038/s41591-021-01622-0

[15] Locke FL , Miklos DB , Jacobson CA , et al. Axicabtagene ciloleucel as second‐line therapy for large B‐cell lymphoma. N Engl J Med. 2022;386(7):640‐654. 34891224 10.1056/NEJMoa2116133

[16] Abramson JS , Solomon SR , Arnason J , et al. Lisocabtagene maraleucel as second‐line therapy for large B‐cell lymphoma: primary analysis of the phase 3 TRANSFORM study. Blood. 2023;141(14):1675‐1684. 36542826 10.1182/blood.2022018730 PMC10646768

[17] Chow VA , Gopal AK , Maloney DG , et al. Outcomes of patients with large B‐cell lymphomas and progressive disease following CD19‐specific CAR T‐cell therapy. Am J Hematol. 2019;94(8):E209‐E213. 31056762 10.1002/ajh.25505 PMC6776079

[18] Atilla PA , Atilla E . Resistance against anti‐CD19 and anti‐BCMA CAR T cells: recent advances and coping strategies. Transl Oncol. 2022;22:101459. 35617812 10.1016/j.tranon.2022.101459 PMC9136177

[19] Rejeski K , Jain MD , Smith EL . Mechanisms of resistance and treatment of relapse after CAR T‐cell therapy for large B‐cell lymphoma and multiple myeloma. Transplant Cell Ther. 2023;29(7):418‐428. 37076102 10.1016/j.jtct.2023.04.007 PMC10330792

[20] Caballero AC , Escribà‐Garcia L , Alvarez‐Fernández C , Briones J . CAR T‐cell therapy predictive response markers in diffuse large B‐cell lymphoma and therapeutic options after CART19 failure. Front Immunol. 2022;13:904497. 35874685 10.3389/fimmu.2022.904497 PMC9299440

[21] Nastoupil LJ , Jain MD , Feng L , et al. Standard‐of‐care axicabtagene ciloleucel for relapsed or refractory large B‐cell lymphoma: results from the US lymphoma CAR T consortium. J Clin Oncol. 2020;38(27):3119‐3128. 32401634 10.1200/JCO.19.02104 PMC7499611

[22] Kwon M , Iacoboni G , Reguera JL , et al. Axicabtagene ciloleucel compared to tisagenlecleucel for the treatment of aggressive B‐cell lymphoma. Haematologica. 2023;108(1):110‐121. 35770532 10.3324/haematol.2022.280805 PMC9827173

[23] Shouse G , Kaempf A , Gordon MJ , et al. A validated composite comorbidity index predicts outcomes of CAR T‐cell therapy in patients with diffuse large B‐cell lymphoma. Blood Adv. 2023;7(14):3516‐3529. 36735393 10.1182/bloodadvances.2022009309 PMC10362276

[24] Jacobson CA , Hunter BD , Redd R , et al. Axicabtagene Ciloleucel in the non‐trial setting: outcomes and correlates of response, resistance, and toxicity. J Clin Oncol. 2020;38(27):3095‐3106. 32667831 10.1200/JCO.19.02103 PMC7499617

[25] Raj S . An inflammatory biomarker signature reproducibly predicts CAR‐T treatment failure in patients with aggressive lymphoma across the zuma trials cohorts. 2023. Available from: https://ash.confex.com/ash/2023/webprogram/Paper173798.html

[26] Vercellino L , Di Blasi R , Kanoun S , et al. Predictive factors of early progression after CAR T‐cell therapy in relapsed/refractory diffuse large B‐cell lymphoma. Blood Adv. 2020;4(22):5607‐5615. 33180899 10.1182/bloodadvances.2020003001 PMC7686887

[27] Dean EA , Mhaskar RS , Lu H , et al. High metabolic tumor volume is associated with decreased efficacy of axicabtagene ciloleucel in large B‐cell lymphoma. Blood Adv. 2020;4(14):3268‐3276. 32702097 10.1182/bloodadvances.2020001900 PMC7391155

[28] Cherng HJJ , Sun R , Sugg B , et al. Risk assessment with low‐pass whole‐genome sequencing of cell‐free DNA before CD19 CAR T‐cell therapy for large B‐cell lymphoma. Blood. 2022;140(5):504‐515. 35512184 10.1182/blood.2022015601 PMC9353148

[29] Strati P , Neelapu SS . CAR‐T failure: beyond antigen loss and T cells. Blood. 2021;137(19):2567‐2568. 33983421 10.1182/blood.2020010462 PMC9635518

[30] Jain MD , Ziccheddu B , Coughlin CA , et al. Whole‐genome sequencing reveals complex genomic features underlying anti‐CD19 CAR T‐cell treatment failures in lymphoma. Blood. 2022;140(5):491‐503. 35476848 10.1182/blood.2021015008 PMC9353150

[31] Shouval R , Alarcon Tomas A , Fein JA , et al. Impact of TP53 genomic alterations in large B‐cell lymphoma treated with CD19‐chimeric antigen receptor T‐cell therapy. J Clin Oncol. 2022;40(4):369‐381. 34860572 10.1200/JCO.21.02143 PMC8797602

[32] Bezombes C , Pérez‐Galán P . Immunotherapies in non‐Hodgkin's lymphoma. Cancer. 2021;13(14):3625. 10.3390/cancers13143625 PMC8305599 34298838

[33] Liu E , Marin D , Banerjee P , et al. Use of CAR‐transduced natural killer cells in CD19‐positive lymphoid tumors. N Engl J Med. 2020;382(6):545‐553. 32023374 10.1056/NEJMoa1910607 PMC7101242

[34] Furqan F , Shah NN . Bispecific CAR T‐cells for B‐cell malignancies. Expert Opin Biol Ther. 2022;22(8):1005‐1015. 35653589 10.1080/14712598.2022.2086043

[35] Furqan F , Shah NN . Multispecific CAR T cells deprive lymphomas of escape via antigen loss. Annu Rev Med. 2023;27(74):279‐291. 10.1146/annurev-med-042921-024719 36332638

[36] Sloas C , Gill S , Klichinsky M . Engineered CAR‐macrophages as adoptive immunotherapies for solid tumors. Front Immunol. 2021;12:783305. 34899748 10.3389/fimmu.2021.783305 PMC8652144

[37] Khurana A , Lin Y . Allogeneic chimeric antigen receptor therapy in lymphoma. Curr Treat Options in Oncol. 2022;23(2):171‐187. 10.1007/s11864-021-00920-6 PMC8873350 35212892

[38] Thomas S , Abken H . CAR T cell therapy becomes CHIC: ‘cytokine help intensified CAR’ T cells. Front Immunol. 2022;13:1090959. 36700225 10.3389/fimmu.2022.1090959 PMC9869021

[39] Iacoboni G , Navarro V , Martín‐López AÁ , et al. Recent Bendamustine treatment before apheresis has a negative impact on outcomes in patients with large B‐cell lymphoma receiving chimeric antigen receptor T‐cell therapy. J Clin Oncol. 2023;42(2):205‐217. 37874957 10.1200/JCO.23.01097

[40] Monfrini C , Stella F , Aragona V , et al. Phenotypic composition of commercial anti‐CD19 CAR T cells affects in vivo expansion and disease response in patients with large B‐cell lymphoma. Clin Cancer Res. 2022;28(15):3378‐3386. 35583610 10.1158/1078-0432.CCR-22-0164 PMC9662896

[41] Roddie C , Neill L , Osborne W , et al. Effective bridging therapy can improve CD19 CAR‐T outcomes while maintaining safety in patients with large B‐cell lymphoma. Blood Adv. 2023;7(12):2872‐2883. 36724512 10.1182/bloodadvances.2022009019 PMC10300297

[42] Bachy E , Le Gouill S , Di Blasi R , et al. A real‐world comparison of tisagenlecleucel and axicabtagene ciloleucel CAR T cells in relapsed or refractory diffuse large B cell lymphoma. Nat Med. 2022;28(10):2145‐2154. 36138152 10.1038/s41591-022-01969-y PMC9556323

[43] Campo E , Swerdlow SH , Harris NL , Pileri S , Stein H , Jaffe ES . The 2008 WHO classification of lymphoid neoplasms and beyond: evolving concepts and practical applications. Blood. 2011;12(117):5019‐5032. 10.1182/blood-2011-01-293050 PMC3109529 21300984

[44] Cheson BD , Fisher RI , Barrington SF , et al. Recommendations for initial evaluation, staging, and response assessment of Hodgkin and non‐Hodgkin lymphoma: the Lugano classification. J Clin Oncol. 2014;32(27):3059‐3068. 25113753 10.1200/JCO.2013.54.8800 PMC4979083

[45] Barrington SF , Mikhaeel NG , Kostakoglu L , et al. Role of imaging in the staging and response assessment of lymphoma: consensus of the international conference on malignant lymphomas imaging working group. J Clin Oncol. 2014;32:3048‐3058. 25113771 10.1200/JCO.2013.53.5229 PMC5015423

[46] Cheson BD . Staging and response assessment in lymphomas: the new Lugano classification. Chin Clin Oncol. 2015;4:5. 25841712 10.3978/j.issn.2304-3865.2014.11.03

[47] Chong EA , Ruella M , Schuster SJ . Five‐year outcomes for refractory B‐cell lymphomas with CAR T‐cell therapy. N Engl J Med. 2021;384(7):673‐674. 33596362 10.1056/NEJMc2030164

[48] Jacobson CA , Munoz J , Sun F , et al. Real‐world outcomes with chimeric antigen receptor T cell therapies in large B cell lymphoma: a systematic review and meta‐analysis. Transplant Cell Ther. 2023;30(1):77.e1‐77.e15. 10.1016/j.jtct.2023.10.017 37890589

[49] Cappell KM , Sherry RM , Yang JC , et al. Long‐term follow‐up of anti‐CD19 chimeric antigen receptor T‐cell therapy. J Clin Oncol. 2020;38(32):3805‐3815. 33021872 10.1200/JCO.20.01467 PMC7655016

[50] Casadei B , Argnani L , Guadagnuolo S , et al. Real world evidence of CAR T‐cell therapies for the treatment of relapsed/refractory B‐cell non‐Hodgkin lymphoma: a monocentric experience. Cancer. 2021;13(19):4789. 10.3390/cancers13194789 PMC8507677 34638273

[51] Di Blasi R , Le Gouill S , Bachy E , et al. Outcomes of patients with aggressive B‐cell lymphoma after failure of anti‐CD19 CAR T‐cell therapy: a DESCAR‐T analysis. Blood. 2022;140(24):2584‐2593. 36122385 10.1182/blood.2022016945

[52] Zurko J , Nizamuddin I , Epperla N , et al. Peri‐CAR‐T practice patterns and survival predictors for all CAR‐T patients and post‐CAR‐T failure in aggressive B‐NHL. Blood Adv. 2023;7(12):2657‐2669. 36094847 10.1182/bloodadvances.2022008240 PMC10333741

[53] Alarcon Tomas A , Fein JA , Fried S , et al. Outcomes of first therapy after CD19‐CAR‐T treatment failure in large B‐cell lymphoma. Leukemia. 2023;37(1):154‐163. 36335261 10.1038/s41375-022-01739-2 PMC9892211

[54] St Martin Y , Franz JK , Agha ME , Lazarus HM . Failure of CAR‐T cell therapy in relapsed and refractory large cell lymphoma and multiple myeloma: an urgent unmet need. Blood Rev. 2023;60:101095. 37173224 10.1016/j.blre.2023.101095

[55] Nastoupil L . Effectiveness of chemo‐immunotherapy (CIT) and novel therapies in second or later line of therapy (2 L+) for patients with relapsed/refractory (R/R) aggressive large B‐Cell lymphoma (LBCL). 2023. Available from: https://ash.confex.com/ash/2023/webprogram/Paper174497.html

[56] Zurko JC , Epperla N , Nizamuddin I , et al. Outcomes and treatment patterns in patients with aggressive B‐cell lymphoma after failure of anti‐CD19 CAR T‐cell therapy. Blood. 2021;138:884.

[57] Pinnix CC , Gunther JR , Dabaja BS , et al. Bridging therapy prior to axicabtagene ciloleucel for relapsed/refractory large B‐cell lymphoma. Blood Adv. 2020;4(13):2871‐2883. 32589728 10.1182/bloodadvances.2020001837 PMC7362355

[58] Lyu C , Cui R , Wang J , et al. Intensive Debulking chemotherapy improves the short‐term and long‐term efficacy of anti‐CD19‐CAR‐T in refractory/relapsed DLBCL with high tumor bulk. Front Oncol. 2021;11:706087. 34395279 10.3389/fonc.2021.706087 PMC8361834

[59] Dickinson M , Carlo‐Stella C , Morschhauser F , et al. Glofitamab in patients with relapsed/refractory (R/R) diffuse large B‐cell lymphoma (DLBCL) and ≥2 prior therapies: pivotal phase II expansion results. J Clin Oncol. 2022;40(16_suppl):7500.

[60] Thieblemont C , Phillips T , Ghesquieres H , et al. Epcoritamab, a novel, subcutaneous CD3xCD20 bispecific T‐cell‐engaging antibody, in relapsed or refractory large B‐cell lymphoma: dose expansion in a phase I/II trial. J Clin Oncol. 2023;41(12):2238‐2247. 36548927 10.1200/JCO.22.01725 PMC10115554

[61] Kim WS . Subcutaneous epcoritamab plus lenalidomide in patients with relapsed/refractory diffuse large B‐cell lymphoma from EPCORE NHL‐5. 2023. Available from: https://ash.confex.com/ash/2023/webprogram/Paper180089.html

[62] Budde LE , Assouline S , Sehn LH , et al. Single‐agent Mosunetuzumab shows durable complete responses in patients with relapsed or refractory B‐cell lymphomas: phase I dose‐escalation study. J Clin Oncol. 2022;40(5):481‐491. 34914545 10.1200/JCO.21.00931 PMC8824395

[63] Ayyappan S . Final analysis of the phase 2 ELM‐2 study: Odronextamab in patients with relapsed/refractory (R/R) diffuse large B‐Cell lymphoma (DLBCL). 2023. Available from: https://ash.confex.com/ash/2023/webprogram/Paper179818.html

[64] Iacoboni G . Efficacy of chimeric antigen receptor T‐Cell therapy is not impaired by previous bispecific antibody treatment in patients with large B‐Cell lymphoma. 2023. Available from: https://ash.confex.com/ash/2023/webprogram/Paper185035.html 10.1182/blood.2024024526 38657242

[65] Crochet G , Iacoboni G , Couturier A , et al. Efficacy of CAR T‐cell therapy is not impaired by previous bispecific antibody treatment in large B‐cell lymphoma. Blood. 2024;144:334‐338. 38657242 10.1182/blood.2024024526

[66] Barba P , Kwon M , Briones J , et al. YTB323 (Rapcabtagene Autoleucel) demonstrates durable efficacy and a manageable safety profile in patients with relapsed/refractory diffuse large B‐cell lymphoma: phase I study update. Blood. 2022;140(Supplement 1):1056‐1059.36074532

[67] Frank MJ , Hossain NM , Bukhari A , et al. Monitoring of circulating tumor DNA improves early relapse detection after Axicabtagene Ciloleucel infusion in large B‐cell lymphoma: results of a prospective multi‐institutional trial. J Clin Oncol. 2021;39(27):3034‐3043. 34133196 10.1200/JCO.21.00377 PMC10166351

[68] Alizadeh A . Circulating tumor DNA dynamics as EARLY outcome predictors for lisocabtagene maraleucel as second‐line therapy for large B‐cell lymphoma from the Phase 3 TRANSFORM study. 2023. Available from: https://ash.confex.com/ash/2023/webprogram/Paper181007.html 10.1182/blood.2022018730 PMC10646768 36542826

[69] Hutchings M . Glofitama monotherapy in relapsed or refractory large B‐Cell lymphoma: extended follow‐up from a pivotal phase II study and subgroup analyses in patients with prior chimeric antigen receptor T‐Cell therapy and by baseline total metabolic tumor volume. 2023. Available from: https://ash.confex.com/ash/2023/webprogram/Paper173951.html

[70] Erbella F , Bachy E , Cartron G , et al. Late failure of aggressive B‐cell lymphoma following CAR T‐cell therapy: a Lysa study from the Descar‐T registry. Blood. 2022;15(140):1325‐1327. 10.1182/bloodadvances.2025016727 PMC12828827 41124667

[71] Bannerji R , Arnason JE , Advani RH , et al. Odronextamab, a human CD20 × CD3 bispecific antibody in patients with CD20‐positive B‐cell malignancies (ELM‐1): results from the relapsed or refractory non‐Hodgkin lymphoma cohort in a single‐arm, multicentre, phase 1 trial. Lancet Haematol. 2022;9(5):e327‐e339. 35366963 10.1016/S2352-3026(22)00072-2 PMC10681157

[72] Brody J . Epcoritamab SC + GemOx leads to high complete metabolic response rates in patients with relapsed/refractory diffuse large B‐cell lymphoma ineligible for autologous stem cell transplant: updated results from Epcore NHL‐2. 2023 Available from: https://ash.confex.com/ash/2023/webprogram/Paper180246.html

